# Evaluation of Antiseizure Activity of Essential Oil from Roots of *Angelica archangelica* Linn. in Mice

**DOI:** 10.4103/0250-474X.70487

**Published:** 2010

**Authors:** Shalini Pathak, M. M. Wanjari, S. K. Jain, M. Tripathi

**Affiliations:** Institute of Pharmacy, Bundelkhand University, Jhansi-284 128, India; 1 Central Research Institute (Ayurveda), Aamkho, Gwalior-474 009, India; Institute of Pharmacy, Bundelkhand University, Jhansi-284 128, India; 2 School of Pharmaceutical Sciences, Rajeev Gandhi Proudyogiki Vishwavidyalaya, Bhopal - 462 036, India

**Keywords:** *Angelica archangelica* (Umbelliferae), convulsions, pentylenetetrazol, medicinal plants, maximal electroshock

## Abstract

In the present study, the effect of essential oil of the root of *Angelica archangelica* Linn. was evaluated against electrically and chemically induced seizures. The seizures were induced in mice by maximal electroshock and pentylenetetrazol. The effect of essential oil of the root of *Angelica archangelica* on seizures was compared with standard anticonvulsant agents, phenytoin and diazepam. The essential oil of the root of *Angelica archangelica* suppressed duration of tonic convulsions and showed recovery in maximal electroshock induced seizures while it delayed time of onset of clonic convulsions and showed mortality protection in pentylenetetrazol induced seizures. The essential oil of the root of *Angelica archangelica* also produced motor impairment at the antiseizure doses. The study indicated that the essential oil exhibited antiseizure effect. The antiseizure effect may be attributed to the presence of terpenes in the essential oil.

*Angelica archangelica* Linn (Umbelliferae) is a perineal herbaceous plant[1] commonly known as *Chorak*. It is cultivated especially for the culinary uses and aromatherapy. It is reported to possess antimutagenic[2], antiulcerogenic[3], hepatoprotective[4], antiproliferative[5] antitumour[6], and cytotoxic[7] effects. Ayurvedic literature documents that *A. archangelica* exhibits significant influence on central nervous system. The essential oil of Angelica sinensis, another species of Angelica, showed the anxiolytic effect[8,9] in animal models.

In Indian system of medicine, *A. archangelica* and especially its roots are used in treatment of epilepsy[10]. Chemical studies have demonstrated that monoterpenes hydrocarbons are present in the essential oil of root of *A. archangelica* (AAO)[11]. Monoterpenes have shown protective effects against pentylenetetrazol-, picrotoxin- and N-methy-D-Aspartate-induced convulsions[12,13]. Further, chemical studies also demonstrated the presence of α-pinene, β-pinene, δ-3-carene, limonene, α-phellandrene, β-phellandrene, p-cymene[11,14] as the major components of the AAO. It is reported that some analogs of pinene prevent the audiogenic seizures in susceptible rats[15,16].

In view of these evidences, it is possible that AAO can exhibit seizure protective effect. The present study was, therefore, undertaken to evaluate the effect of AAO on experimentally induced seizures. Moreover, it was also assessed whether, at the anticonvulsant doses, AAO causes motor impairment.

The essential oil of root of *A. archangelica* (AAO) was obtained from Rakesh Industries, Kanpur, India. Phenytoin sodium was procured as gift sample from Anglo-French Drugs and Industries, Bengaluru while diazepam was procured from local pharmacy (Calmpose). Pentylenetetrazol was purchased from Sigma chemicals, USA. For administration purpose, AAO was diluted with arachis oil to obtain the desired doses. The other drugs were prepared freshly by dissolving in normal saline. All the drugs and AAO were injected intraperitoneally (i.p.).

Albino Swiss mice (20-30 g) of either sex (procured from Central Drug Research Institute, Lucknow, India) maintained in Central Animal Facility of the Institute were used throughout the study. The animals were maintained at constant room temperature (22±2°) and 12-h light/12-h dark cycle with food and water *ad libitum*. The experimental protocols were approved by the Institutional Animal Ethical Committee constituted for the control and supervision on experiments on animals.

The authenticity of the AAO was assessed by determining its physicochemical characteristics viz. refractive index, specific gravity, acid value and boiling point by standard conventional methods.

Acute toxicity study was performed in mice according to Staircase method. The dose was increased from 500 mg/kg to 2000 mg/kg through i.p. route (5 animals per dose). The mice were observed individually after dosing at least once during the first 30 min periodically during the first 24 h, with special attention given during the first 4h.

In Maximal electroshock (MES) induced seizures,[17] an electroconvulsive shock (50 mA for 0.2 sec) was delivered through corneal-electrode to induce hind limb tonic extensor phase (HLTE) in mice. Prior to delivery, the current output was checked by Multimeter. The current was delivered to 6 groups of mice (n=6) 30 min after i.p. administration of arachis oil (10 ml/kg) or AAO (50, 100, 200, 400 mg/kg) or phenytoin sodium (25 mg/kg). After the electric shock, the occurrence and duration of HLTE and incidence of mortality were noted. The animals that did not exhibit HLTE and death were considered protected.

In pentylenetetrazol (PTZ)-induced seizures[17], PTZ (80 mg/kg, CD_99_ dose) was injected i.p. to induce general clonic convulsions in mice. After PTZ injection, the mice were observed for onset and duration of general clonus and mortality. PTZ was administered to 7 groups of mice (n = 6) 30 min after i.p. administration of arachis oil (10 ml/kg) or AAO (50, 100, 200, 400, 500 mg/kg) or diazepam (4 mg/kg). If no general clonus occurred, the animal were considered protected.

The rota-rod test[18] was used to determine the effect of AAO on motor incordination. Mice were placed on horizontal metal-coated rod (2.5 cm diameter) rotating at speed of 22 rpm. The time, each mouse was able to maintain its balance walking on top of the rod, was measured and cut off time was kept 300 sec. Before the beginning of all experiments, the riding ability of the animals on rota-rod was checked. Thus, the mice were initially put on a rotating rod, and mice that immediately dropped off (within 60 sec.) were excluded from the experiment. The test was conducted on 7 groups (n = 6) of previously screened mice, 30 min after the injection of arachis oil (10 ml/kg) or AAO (50, 100, 200, 400, 500 mg/kg) or diazepam (4 mg/kg).

The traction test[19] was also used to determine the effect of AAO on motor incordination. Forepaws of a mouse were placed on a 15 cm long twisted wire rigidly supported and 20 cm above the table top. Normal mice grasped the wire with forepaws and when allowed to hang free, placed at least one hind foot on the wire within 5 sec. Inability to put up at lest one hind foot was considered failure to the traction. The test was conducted on 7 groups (n = 6) previously screened mice, 30 min after the injection of arachis oil (10 ml/kg) or AAO (50, 100, 200, 400, 500 mg/kg) or diazepam (4 mg/kg).

The data from MES and PTZ test were analyzed by Chi Square test for the assessment of protection of convulsion. The ED_50_ and TD_50_ values were calculated by Litchfield and Wilcoxan method. The data from traction test were analyzed by Chi Square test while the data from rota rod test were analyzed by one way ANOVA followed by Dunnett multiple comparisons test. A difference of *p*<0.05 was considered significant in all cases.

The assessment of physicochemical parameters showed that AAO exhibited light greenish yellow colour, strong aromatic odour, refractive index (1.46900-1.47800), specific gravity (0.85000-0.88000), boiling point (82±2°), and acid value (4.0-4.2). These values were similar to those reported earlier[1] indicating the authenticity of the AAO.

The toxicity studies revealed that maximum tolerable dose for AAO was more than 2000 mg/kg. No signs of toxicity or moribund state were found at all the dose of AAO tested. Therefore, the approximate LD_50_ is more than 2000 mg/kg.

The Chi-square analysis indicated that AAO treatment exhibited significant antiseizure activity [(df= 24.79, 5), *p*<0.05] (Table 1) against MES-induced seizure with an ED_50_ of 373.53 mg/kg. The AAO also prevented clonic seizures induced by PTZ (*p*<0.05, df= 29.36, 6, Table 2) with an ED_50_ value 214.62 mg/kg for the AAO. Phenytoin and diazepam (positive controls) produced 100% protection against seizures induced by MES and PTZ.

**TABLE 1:** EFFECT OF AAO ON MAXIMAL ELECTROSHOCK INDUCED SEIZURES IN MICE

Treatments	Duration of HLTE (sec)	% protection	% mortality
Vehicle	11.34±0.1357	0	100
Phenytoin	0.0	100	0
AAO 50	9.468±0.1548	0	83.34
AAO 100	8.355±0.09915*	0	100
AAO 200	5.223±0.1184*	0	66.67
AAO 400	0.0	100	0

* *p*<0.05 compared to vehicle control (Chi-square test). Separate groups of mice were injected with vehicle (10 ml/kg, i.p.) or phenytoin sodium (25 mg/kg, i.p.) or increasing doses of AAO (50-400 mg/kg, i.p.), and 30 min thereafter the current was delivered to each mouse. Immediately after electrical stimulation individual mouse was tested for the occurrence and duration of HLTE and incidence of mortality. Each value represents mean±SEM of data from 5-6 mice. *p<0.05 vs. respective vehicle control (Chi-square test).

**TABLE 2:** EFFECT OF AAO ON PENTYLENETETRAZOL INDUCED SEIZURES IN MICE

Treatments	Onset (sec)	Duration (sec)	% protection	% mortality
Vehicle	74±1.72	198±5.40	0	100
Diazepam	----	-----	100	0
AAO 50	67.83±3.80	170.8±6.0	0	100
AAO 100	61.00±6.86	166.2±18.59	0	100
AAO 200	80.00±5.19	182.3±9.72	0	100
AAO 400	245.7±25.27*	12.67±3.18*	50	50
AAO 500	282.0±0*	8.000±0*	83.33	16.6

* *p*<0.05 compared to vehicle control (Chi-square test). Separate groups of mice were injected with vehicle (10 ml/kg, i.p.) or diazepam (4 mg/kg, i.p.) or increasing doses of AAO (50-500 mg/kg, i.p.), and 30 min thereafter PTZ was administered individual mouse. Immediately, the mice were observed for onset and duration of general clonus and mortality. Each value represents mean±SEM of data from 5-6 mice. *p<0.05 vs. respective vehicle control (Chi-square test).

In traction test, the Chi-square analysis indicated that, the AAO exhibited significant (*p*<0.05, df= 25.33, 6) sedation and motor impairment. AAO demonstrated dose related decrease in the time of holding compared to vehicle. AAO 100, 200 and 400 mg/kg showed 20, 20 and 80% failure respectively and AAO 500 mg/kg indicated 100% failure to put at least one hind limb on wire Thus, the TD_50_ of AAO in traction test was found to be 277.27 mg/kg. In rota- rod test, one-way ANOVA showed that AAO has significant influence on the motor function (*p*<0.05). Dunnett’s multiple comparisons indicated AAO showed a significant decline (*p*<0.05) in the motor function maximum at the doses which produced antiseizure effect (fig. 1).

**Fig. 1:**
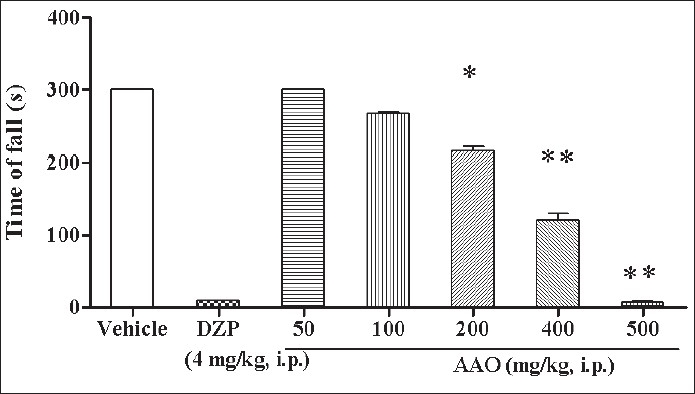
Effect of AAO on rota rod test Separate groups of mice were injected with vehicle (10 ml/kg, i.p.) or diazepam (4 mg/kg, i.p.) or increasing doses of AAO (50-500 mg/kg, i.p.), and 30 min thereafter motor incordination was tested on rota rod. Each bar represents mean±SEM of data from 5-6 mice. FNx01*p*<0.05 vs. respective vehicle control and Diazepam control (one-way ANOVA followed by post hoc Dunnett test).

The MES test is used primarily as an indication for compounds which are effective in grand mal epilepsy while the PTZ test represents a valid model for human generalized myoclonic and also absence seizures[20]. The present study revealed that the essential oil of the root of *Angelica archangelica* attenuated both MES-induced tonic and PTZ-induced clonic seizures indicating that AAO possesses antiseizure activity. These findings corroborate well with a recent study that demonstrated the anticonvulsant effect of imperatorin (a furanocoumarin isolated from fruits of *Angelica archangelica*) in the mouse maximal electroshock seizure threshold model[21] and support the antiseizure activity *Angelica archangelica*. However, it is not clear that how does AAO exhibit antiseizure activity.

As mentioned earlier, AAO contains more than 60% monoterpenes[11] and monoterpenes have been found to show protective effects against PTZ, picrotoxin-and NMDA-induced convulsions[12,13]. Modulations of glutamatergic and GABAergic transmission are mechanisms indicated for anticonvulsant action of the monoterpenes[13,22,23]. Therefore, it is possible that the antiseizure effect of AAO can be due to the presence of monoterpenes present in AAO and subsequent modulation of glutamatergic and GABAergic transmission.

α-pinene is one of the major constituent of AAO[11]. It is reported that some analogs of pinene prevent the audiogenic seizures in susceptible rats[15] which further strengthens the anti-seizure potential of AAO and it may be related to terpenoids present in the oil. The investigations further revealed that AAO produced motor impairment at antiseizure doses. Some terpenes such as eugenol and anethol have anesthetic, sedative and muscle relaxant effects[24,25]. The terpenes present in the essential oil may be responsible for the observed motor impairment subsequent to CNS depression. Thus, the present investigations conclude that AAO exhibits significant antiseizure activity against chemically and electrically induced seizures in mice and the same can be attributed to terpenes especially monoterpenes present in the Angelica essential oil.
